# Vocational Rehabilitation in Mild Traumatic Brain Injury: Supporting Return to Work and Daily Life Functioning

**DOI:** 10.3389/fneur.2019.00103

**Published:** 2019-02-21

**Authors:** Frederik Lehman Dornonville de la Cour, Morten Arendt Rasmussen, Eva Meldal Foged, Line Svenning Jensen, Trine Schow

**Affiliations:** ^1^Research and Development, Brain Injury Center BOMI, Roskilde, Denmark; ^2^Department of Psychology, Faculty of Health Sciences, University of Southern Denmark, Odense, Denmark; ^3^Copenhagen Prospective Studies on Asthma in Childhood (COPSAC), Herlev and Gentofte Hospital, University of Copenhagen, Copenhagen, Denmark

**Keywords:** mild traumatic brain injury, concussion, post-concussive syndrome, vocational rehabilitation, multidisciplinary rehabilitation, return to work, employment, standard operating procedure (SOP)

## Abstract

Persisting post-concussive symptoms are challenging to treat and may delay return-to-work (RTW). The aims of this study were to describe a multidisciplinary and holistic vocational rehabilitation (VR) program for individuals with mild traumatic brain injury (mTBI) and to explore course and predictors of employment outcome during VR. The VR program was described using the Standard Operating Procedures (SOPs) framework. Further, a retrospective, cohort study on individuals with mTBI receiving VR was conducted based on clinical records (*n* = 32; 22% males; mean age 43.2 years; 1.2 years since injury on average). The primary outcome was difference in hours at work per week from pre- to post-VR, and the secondary outcome was change in a three-level RTW-status. Time since injury, age, sex, and loss of consciousness were investigated as predictors of the outcomes. The VR intervention is individually tailored and targets patients' individual needs. Thus, it may combine a variety of methods based on a biopsychosocial theoretical model. During VR, hours at work, 17.0 ± 2.2, *p* < 0.001, and RTW-status, OR = 14.0, *p* < 0.001, improved significantly with 97% having returned to work after VR. Shorter length of time since injury and male sex were identified as predictors of a greater gain of working hours. Time since injury was the strongest predictor; double the time was associated with a reduction in effect by 4.2 ± 1.4 h after adjusting for working hours at start of VR. In sum, these results suggest that individuals facing persistent problems following mTBI may still improve employment outcomes and RTW after receiving this multidisciplinary and holistic VR intervention, even years after injury. While results are preliminary and subject to bias due to the lack of a control group, this study warrants further research into employment outcomes and VR following mTBI, including who may benefit the most from treatment.

## Introduction

Traumatic brain injury (TBI) is a significant cause of morbidity and mortality worldwide and constitutes the third largest health expense in the USA (1, 2). The vast majority of cases (70–90%) are categorized as mild TBI (mTBI), or concussion (3). Individuals with mTBI tend to show considerable variation in post-concussive symptoms, which may include headache, fatigue, vestibular, and vision dysfunctions, increased sensitivity to light, noise, and pain, vertigo, sleep disturbances, cognitive deficits such as reduced concentration and poor memory, or mental health issues (4, 5). Most individuals sustaining mTBI recover spontaneously during the first week, however, a small subset continue to experience persisting symptoms beyond 3 months post-injury (6) with long-term implications for vocational, recreational, and social activities (7). Some individuals may even experience symptoms for more than 1 year after the incident (8). Persisting symptoms may delay return-to-work (RTW), reduce work productivity, adversely affect quality of life, and result in additional social and economic costs.

Evidence on vocational outcomes following mTBI is limited, and rates of RTW vary widely between studies (9, 10). Results of a systematic review suggest that most workers RTW within 3–6 months after mTBI, however, 5–20% continue to experience work limitations for 1–2 years post-injury (9), and possibly even longer (11). RTW is often associated with increased psychological well-being and quality of life (12), and is thus often identified as a major goal of recovery. However, even when returning to work, some individuals still experience distressing post-concussive symptoms, suffer from comorbid psychiatric conditions such as depression and anxiety, and work with functional limitations and reduced productivity (13). Further, individuals with mTBI may experience challenges maintaining employment over time.

Employment outcomes following mTBI can be complicated by multiple factors, including personal, injury-related, and environmental factors. Research regarding specific predictors of outcome such as age, sex, or various injury-related factors is mainly inconclusive (14). Some evidence suggests that a lower level of education, nausea or vomitting on hospital admission, extracranial injuries, severe pain early after injury, and limited job independence and decision-making latitude predict delayed RTW (9). Wäljas et al. (15) identified age, multiple bodily injuries, intracranial abnormality, and fatigue as predictors of delayed RTW, and Vikane et al. (16) reported psychological distress, global functioning post-injury, and being sick-listed 2 months after and the last year before mTBI as predictors. Looking more specifically at productivity loss, Silverberg et al. (13) found that residual symptoms and comorbid psychiatric conditions were predictors, and regarding long-term outcomes, Theadom et al. (11) reported that cognitive complaints at 1 month post-injury were predictive of work limitations 4 years post-injury. A recent systematic review supports the role of cognition in predicting and facilitating RTW (17). Thus, a range of factors, including demographic, physical, cognitive, and emotional as well as environmental and societal, may impact the course of employment outcomes after mTBI in a complex interaction, which is yet unclear.

Treatment of persistent symptoms after mTBI is based on limited evidence (18), and so is vocational rehabilitation (VR) more specifically (19). VR can broadly be defined as “whatever helps someone with a health problem to stay at, return to and remain in work” (20) and may require a combination of healthcare and workplace interventions. Regarding mTBI, there is clinical concensus that recommendations should be individually tailored and based on a multidisciplinary evaluation of personal, environmental, and occupational factors (21). Thus, VR constitutes a combination of individually tailored approaches; from initial assessment through intervention to evaluation of the patient's progress. Examples of means of promoting RTW and improving employment outcomes may be to reduce and in turn, if possible, gradually increase weekly working hours, to modify job demands, tasks, and the work environment, and to introduce rest breaks during the work day (19).

VR, like other interventions within rehabilitation, lacks definitions of treatment approaches. Definition and development of treatment manuals within neurorehabilitation have been debated comprehensively in the literature for more than a decade. However, there is still no clear-cut recipe or right or wrong way of how to develop an efficient treatment manual in this complex and multidisciplinary field of treatment, where interventions involve a variety of different methods. In designing a manual, one has to balance between how rigid vs. flexible, how long vs. short, and how detailed vs. broad to make the manual, all depending on the context, in which it is to be used, and the nature of the treatment itself (22–26).

Previous research has primarily investigated the course of RTW following mTBI, and only few studies investigated the course and predictors of more detailed employment outcomes in individuals with mTBI undergoing VR. Further, contents and strategies of VR for mTBI are seldom described in detail. This study aimed to describe a multidisciplinary and holistic VR program for individuals with persisting post-concussive symptoms. Further, the study aimed to compare employment outcomes in individuals with mTBI before and after completing the VR program. It was hypothesized that participants work more hours per week following the VR program. Finally, the study aimed to investigate a panel of four baseline characteristics as predictors of employment outcomes in an exploratory analysis. Apriory, time since injury was considered the most influencial factor, then secondary age, sex, and loss of consciousness in parallel.

## Materials and Methods

### Design and Setting

This study was conducted at the specialized brain injury center BOMI in Denmark. BOMI offers multidisciplinary and individually tailored VR for individuals with brain injury, including mTBI and comorbid conditions. First, the Standard Operating Procedures (SOPs) framework was used to describe the VR program for mTBI. Second, a retrospective cohort study was conducted based on clinical records.

### Development of Standard Operating Procedures

Aims, contents, and procedures for each module of the VR program were described in an intervention protocol using the SOPs framework (27). SOPs are specific standardized procedures that regulate the routine actions of individuals in specific positions and assign roles and responsibilities. SOPs within neurorehabilitation can act as a local adaptation of clinical guidelines (if such exists), based upon evidence-based practice. Implementation of guidelines in clinical practice often requires adaptation by the local workplace, where the guideline recommendations are combined with expert knowledge and routines. SOPs will help bridge the gap between evidence-based medicine (clinical guidelines) and the local circumstances and possibilities for carrying out rehabilitation. The SOP guides both the experienced and the inexperienced therapist through the same decision making processes to support a goal-oriented manner of practice.

For development of the SOPs, two representatives of each professional group in the multidisciplinary team providing VR at BOMI were recruited. That is, two occupational therapists, two physiotherapists, and two neuropsychologists. To be included, professionals had to be skilled with VR; hence, they had to have at least 2 years of experience with VR at BOMI. The staff members participated in workshops to discuss theory, goals, effective components, and practical approaches of VR.

### Cohort Study

#### Participants

BOMI Center for Rehabilitation and Brain Injury receives individuals with acquired brain injury from a large number of Danish municipalities, primarily from the Capital and Zealand regions of Denmark. Since 2011, one municipality from the Capital region has consistently referred all individuals, who require treatment for persisting symptoms following mTBI, to receive multidisciplinary treatment and VR at BOMI. For this study, we included all individuals with an mTBI diagnosis from this municipality, who had received VR at BOMI between 2011 and 2018. Individuals are referred to BOMI as soon as they report problems that involve sick leave from work for more than 1 month or a need to take a sick leave after struggling with symptoms for several months. Consequently, time since injury may vary among referred individuals. Individuals have not necessarily been hospitalized for their mTBI. In the beginning of this collaboration, the municipality did not identify as many individuals with mTBI as in the later years. The identification procedures needed to be implemented throughout different levels in the organization of the municipality where moderate to severe TBI previously were prioritized. However, during the years, the procedures of how to identify individuals with persisting symptoms after mTBI became more clear and the number of referred individuals with mTBI increased.

Clinical records at BOMI were screened to confirm that participants of this study had been exposed to a trauma involving a direct blow to the head or involving a coup-contrecoup movement. Further, participants had to fulfill at least one of the following criteria: Loss of consciousness (max. 30 min), post-traumatic amnesia for a period of max. 24 hours, disturbance of consciousness (confusion or disorientation in time, place, or personal data), or transient neurological symptoms. In addition, participants had to have a Glasgow Coma Scale score above 13 after 30 min. All participants completed the planned rehabilitation program.

#### Measures

Data was collected from clinical records and chart reviews. Pre-injury data was self-reported retrospectively at start of VR.

##### Demographics and injury-related factors

Demographics were recorded, including sex, age, educational level, living arrangement, and number of children. The following injury-related data was recorded: Time since injury, the event causing injury, loss of consciousness at injury, and earlier incidents of concussion. Finally, duration of VR was recorded. The duration of VR depended on a variety of factors, including the patients' progress and needs and the financial frame granted by the municipality.

##### Employment outcomes

Four indicators of employment outcome were evaluated: Hours at work per week, RTW, full-time vs. part-time work, and employment status. The number of hours at work or education (high school, college, or university level) was recorded for three time points: At time of injury (T_1_), at start of VR (T_2_), and at completion of VR (T_3_). RTW was evaluated at pre- (T_2_) and post-VR (T_3_). RTW was divided into complete and partial RTW by comparison with working hours at time of injury (T_1_). That is, complete RTW corresponds to returning to the same (or an increased) amount of hours per week compared to pre-injury, and partial RTW corresponds to returning to a reduced amount of hours. Full-time work was defined as ≥30 productive hours per week and part-time as 0 <30. Finally, employment status was evaluated as competitive employment, supported employment, or sick leave.

#### Intervention

All participants received individually tailored, face-to-face, multidisciplinary VR. Details of the program are described in the Results section.

#### Analyses

Demographics, injury-related variables and employment outcomes were explored using descriptive statistics. The primary outcome was defined as the difference in working hours before and after VR. This outcome was evaluated by linear models. The secondary outcome was RTW with three levels (i.e., complete RTW, partial RTW, and no RTW) and was treated as an ordinal outcome. This outcome was evaluated by ordinal regression. For both outcomes, four variables were investigated as predictors: time since injury, age, sex, and loss of consciousness. They were investigated univariately using simple linear models with either categorical or continuous variables as predictors, reporting relevant effect sizes. Initial inspection of data revealed that the distribution of data for “time since injury” differed from being normally distributed, and this variable was hence log2 transformed before the main analyses. The statistical analyses were conducted in R version 3.4.2 (28) using describe() and stat.desc() from the packages psych and pastecs, respectively, for descriptive statistics, ggplot2 for plotting, base lm() and glm() for linear models and clm() from the ordinal package for ordinal regression. For mixed effect longitudinal models, lmer() from lme4 was used.

#### Ethics

The study was conducted in concordance with the Declaration of Helsinki, and the database was approved by the Danish Data Protection Agency (J.no. 2017-41-5256).

## Results

### A Multidisciplinary Vocational Rehabilitation Program

In this section, the SOPs for VR of concussion are described in headings offering an overview of its content. The SOP theoretical foundation is based on a biopsychosocial theoretical model and the hypothesis that post-concussion symptoms probably represent the cumulative effect of multiple variables such as trauma severity, genetics, mental health history, current life stress, general medical problems, chronic pain, depression, social problems, and personality. Thus, a large variety of cause-effect interactions may contribute to the symptoms, and a full description is therefore not included in this paper.

The SOPs are nested in a circular process, aiming at a continuous evaluation of a patient's progress and responses to treatment. The therapist begins by setting goals for the patient based on an initial analysis of the patient's symptoms and a hypothesis on the underlying causes. Then the therapist chooses a strategy of how to reach the goals, by reanalyzing the patient's state according to goals and treatment. The therapist relates the choices of action according to the hypothesis of underlying causes to the patient's problems, and adjusts the goals and intervention according to the continuous observation.

The VR program for mTBI is individually tailored for each patient and consists of different modules that address the patient's symptoms. Each module has the overall purpose of supporting the patient's workability, either in a direct or more in-direct manner. The combination, length, and intensity of the modules are determined based on the patient's situation, goals for the intervention, and the financial frame granted by the municipality.

The purpose of the concussion VR intervention is:

1. To delineate a holistic understanding of the patient's functioning and disability, and the individual factors involved, including assessment of fatigue, sleep disorders, headache, cognitive difficulties, visual and balance problems, mental health and coping strategies.
2. Supporting that the patient achieves a balance between home life, family life, leisure life, and working life so the patient can participate in necessary and desirable activities and roles.
3. To support the improvement of individual workability, and to allow the patient to RTW as soon and at as many hours per week as possible.

#### Ad1: Assessment and Analysis

First step in the VR intervention is to set goals for the intervention process and patient progress. Typical goals may consist of: Increased insight into different aspects of brain injury and its implications, goals of handling fatigue, incorporation of positive everyday routines to increase energy level throughout the day and prioritize desirable activities, scheduling and planning activities, goals of how to handle cognitive difficulties, monitoring own progress, and reflection of achieved functions.

Throughout this process, therapists collaborate with a neuropsychologist in order to continuously adjust the strategies to each patient's individual cognitive and psychological state.

#### Ad2: Individually Tailored Intervention

Second step is to plan intervention by setting up a hypothesis of the desired change in patient's physical, cognitive, mental, and/or behavioral state in order to reach the goal based on previous evaluations. Thus, the treatment must be somehow broad in methodology to incorporate an approach matching each patient's needs, goals, and circumstances.

Most of the intervention involves change of behavior and adapting compensational strategies. These strategies contribute to teaching the patient to manage different symptoms and daily living in a more appropriate way and initiate a positive lifecycle. The choice of modules, including the length and intensity of modules, all depend on the patient's symptoms and response to intervention. Modules may include:

##### Energy management (EM)

The therapist supports the patient in testing and implementing strategies of how to change routines and amount of daily activities so the patient's energy level will remain stable throughout the day. EM is a personal process where the therapist acts as a facilitator and coach. This involves supporting the patient to set up realistic goals for the energy management process involving that the patient works with: Habits, routines and ways of thinking, life values, family roles and identity, how to interact with others, and more.

Specific approaches in EM may be: Small breaks, breaks at fixed time points, midday nap, ensuring a good night sleep by introducing good sleep hygiene, testing need for ball blanket, use of mindfulness techniques, relaxation techniques, analyzing eating habits and implementing a healthy diet, performing exercise, and achieving positive experiences.

The therapist continuously follows the patient's energy level throughout the day, to help the patient adjust working hours, activity planning, adjusting according to surroundings and other personal or environmental factors both at work and at home. The occupational therapist is in charge of the EM approach in close collaboration with the neuropsychologist.

##### Neuropsychological intervention

The focus of the neuropsychologist is psychoeducation, involving reflection on the patient's thinking patterns regarding new life circumstances, depreciation of the symptoms, and anxiety and depression management. The neuropsychologist conducts an assessment of the psychological status, including symptoms and severity of depression, post-traumatic stress disorder, and anxiety. Furthermore, an evaluation of subjective cognitive level of functioning is conducted using an interview. Based on the psychological evaluation, the patient is offered individually adapted psychotherapy consisting of 3–30 sessions in which the patient is informed about the psychological and cognitive level of functioning, the interrelations of cognitive and psychological functions, thinking and behavioral patterns, and emotional reactions. Different compensation strategies are discussed and developed. Furthermore, existential dilemmas regarding new life circumstances such as health anxiety, relations, being in the world with new physical circumstances, altered time and space, and financial concerns are addressed.

##### Visual and balance training

Another key component of concussion VR is visual and balance training. This training is provided by a team of optometrists and physical therapists. The training involves individualized sensory integration, vestibular and proprioceptive exercises in combination with binocularity, fixation, tracking, vergence, and eye-hand coordination (29). Typically, the patient receives 16–23 weekly sessions (or every other week), depending on the severity of symptoms and responses to the exercises. This training also involves instructions of how to implement exercises and symptom management strategies in everyday activities and work.

##### Physiotherapy

This treatment focuses on dizziness, balance problems, neck problems, pain, and headache. The training is performed individually and is often supported by home-exercises. The principles revolve around graduated exercise training, e.g., focusing on vestibular rehabilitation, active treatment on cervical spine, dynamic stability, adjusted according to pain and progress. The training always involves instructions of how to implement exercises and training in everyday activities. If severe neck problems are suspected, the patient is referred to a physical therapist specialist with a certification in neck problems. Further, if vision problems are suspected, the patient is referred to neuro-optometrist for visual assessment followed by interdisciplinary visual and balance training.

##### Mindfulness

The approach of mindfulness at BOMI is primarily based on “Mindfulness Based Stress Reduction” and, in addition, is inspired by “Mindfulness Empathy and Cohesion.” The purpose is to help the patient gain increased focus on sensitivity, indulgence, self-care, and awareness. As for the other modules, specific techniques are individually planned according to the needs of patients. Exercises may include “body scan,” sitting and/or walking meditation, breathing exercises, and gentle yoga with mindfulness of movements and bodily sensations.

#### Ad3: Vocational Support

In VR, it is recommended that the patient start on a low amount of working hours and a minimum amount of tasks. Thus, the patient typically commences with a few hours at work a day, few days a week, and with a low complexity in work tasks.

There is a close monitoring process of the patient's symptoms, and adjustment of hours at work and work tasks, to ensure that the total work load matches each patient's condition and energy level at work and at home. The therapist will usually see the patient once a week in the beginning, depending on the complexity and patient needs, whilst the frequency and intensity of contact decreases over time. The therapist may also act as a safety net for the patient. Thus, the patient is encouraged to contact the therapist outside of scheduled sessions if needed. The therapist has the authority to contact other relevant personnel, if necessary.

The therapist visits the patient's workplace to analyze and assess compensational strategies and need of work place adjustments. The assessment consists of the combination of subjective information (what the therapist is told by the workplace) and objective information (what the therapist observes at the workplace), and is continuously revised during workplace meetings and during individual contact with the patient. Relevant compensational strategies vary from patient to patient and depend on the patient's difficulties and resources. Compensational strategies involves support related to: When and how the patient should take breaks during work, how the patient compensates for difficulties in forming and maintaining an overview of work tasks, as well as planning different work tasks.

Based on the compensational strategy analysis, the therapist and the patient have reflective conversations in order to help patients evaluate their difficulties and resources. This involves discussions of the linkage between difficulties at the work place and the brain injury, how to use selected compensational strategies, the purpose of incorporating positive working routines and reflection on the individual goals.

### Cohort Study

#### Characteristics of the Cohort

Thirty-two participants were included in the cohort. Mean age at start of VR was 43 years (*SD* = 11; range 18–65 years), and 78% of the participants were female. The majority of participants were living with a partner (59%), 28% were living alone, and 9% were living with parents. Most participants (77%) had children with a median amount of 2 (IQR = 1).

Median number of days since injury was 195 (*IQR* = 273; range = 77–2,030) at start of VR. Duration of VR varied from 97 to 778 days with a median amount of 366 days (*IQR* = 218). Incidents of injury included a fall (34%), a traffic accident (34%), sports-related injuries and injuries due to a blow to the head (31%). The minority of participants had been unconscious following the incident (22%). Please see Table 1 for an overview of participant characteristics and Table 2 for an overview of employment outcomes at pre-injury, pre-VR, and post-VR, respectively.

**Table 1:** Characteristics of the cohort.

**Variable**	**Statistic**	**Participants (*N* = 32)**
**DEMOGRAPHICS**		
Age^a^, years	*M* (*SD*)	43.2 (11.1)
**Sex**		
Male	*n* (%)	7 (22%)
Female	*n* (%)	25 (78%)
**Educational level, years of education**		
0 ≤ 10	*n* (%)	3
11–13	*n* (%)	4
>13	*n* (%)	25
**Living arrangement**		
Cohabiting	*n* (%)	19 (59%)
Living alone	*n* (%)	9 (28%)
Living with parents	*n* (%)	3 (9%)
Missing data	*n* (%)	1 (3%)
**INJURY FACTORS**		
**Cause of injury**		
Fall	*n* (%)	11 (34%)
Traffic accident	*n* (%)	11 (34%)
Sports-related/blow to head	*n* (%)	10 (31%)
**Loss of consciousness**		
No	*n* (%)	25 (78%)
Yes	*n* (%)	7 (22%)
Time since injury^a^, days	*M* (*SD*)	418.66 (531.8)
	*Mdn* (*IQR*)	195 (237.3)
Time since injury after VR, days	*M* (*SD*)	785.81 (511.2)
	*Mdn* (*IQR*)	637.48 (206.5)
**Earlier incidence of concussion**		
No	*n* (%)	29 (91%)
Yes	*n* (%)	3 (9%)
**TREATMENT FACTORS**		
Duration of VR, days	*M* (*SD*)	367.16 (158.7)
	*Mdn* (*IQR*)	366 (218)

*M, mean; Mdn, median; VR, vocational rehabilitation*.

a *At start of VR*.

**Table 2:** Employment outcomes at time of injury, at start of VR, and after VR.

**Variable**	**Statistic**	**Pre-injury (T_**1**_)**	**Pre-VR (T_**2**_)**	**Post-VR (T_**3**_)**
Hours at work per week	*M* (*SD*)	33.7 (10.0)	10.2 (10.4)	27.1 (10.8)
	*Mdn* (*IQR*)	37 (0.5)	9 (16.5)	30 (17.5)
**RTW-STATUS**				
Complete RTW	*n* (%)	–	2 (6%)	14 (44%)
Partial RTW	*n* (%)	–	18 (56%)	17 (53%)
No RTW	*n* (%)	–	12 (38%)	1 (3%)
**WORKING TIME**				
Full-time (≥30 h)	*n* (%)	29 (91%)	3 (9%)	18 (56%)
Part-time (1–29 h)	*n* (%)	1 (3%)	17 (53%)	13 (41%)
No work (0 h)	*n* (%)	2 (6%)	12 (38%)	1 (3%)
**EMPLOYMENT STATUS**				
Competitive employment	*n* (%)	30 (94%)	15 (47%)	21 (66%)
Supported employment	*n* (%)	0 (0%)	1 (3%)	8 (25%)
Sick leave	*n* (%)	0 (0%)	15 (47%)	1 (3%)
Other^a^	*n* (%)	2 (6%)	1 (3%)	2 (6%)

*Complete RTW represents working the same (or an increased) amount of hours compared to pre-injury, partial RTW represents working fewer hours compared to pre-injury, and no RTW represents not working any hours per week. VR, vocational rehabilitation; M, mean; Mdn, median; RTW, return-to-work*.

a *Other includes unemployment and non-competitive work/non-payed work trials*.

#### Differences in Employment Outcomes Before and After Vocational Rehabilitation

**Figure 1:**
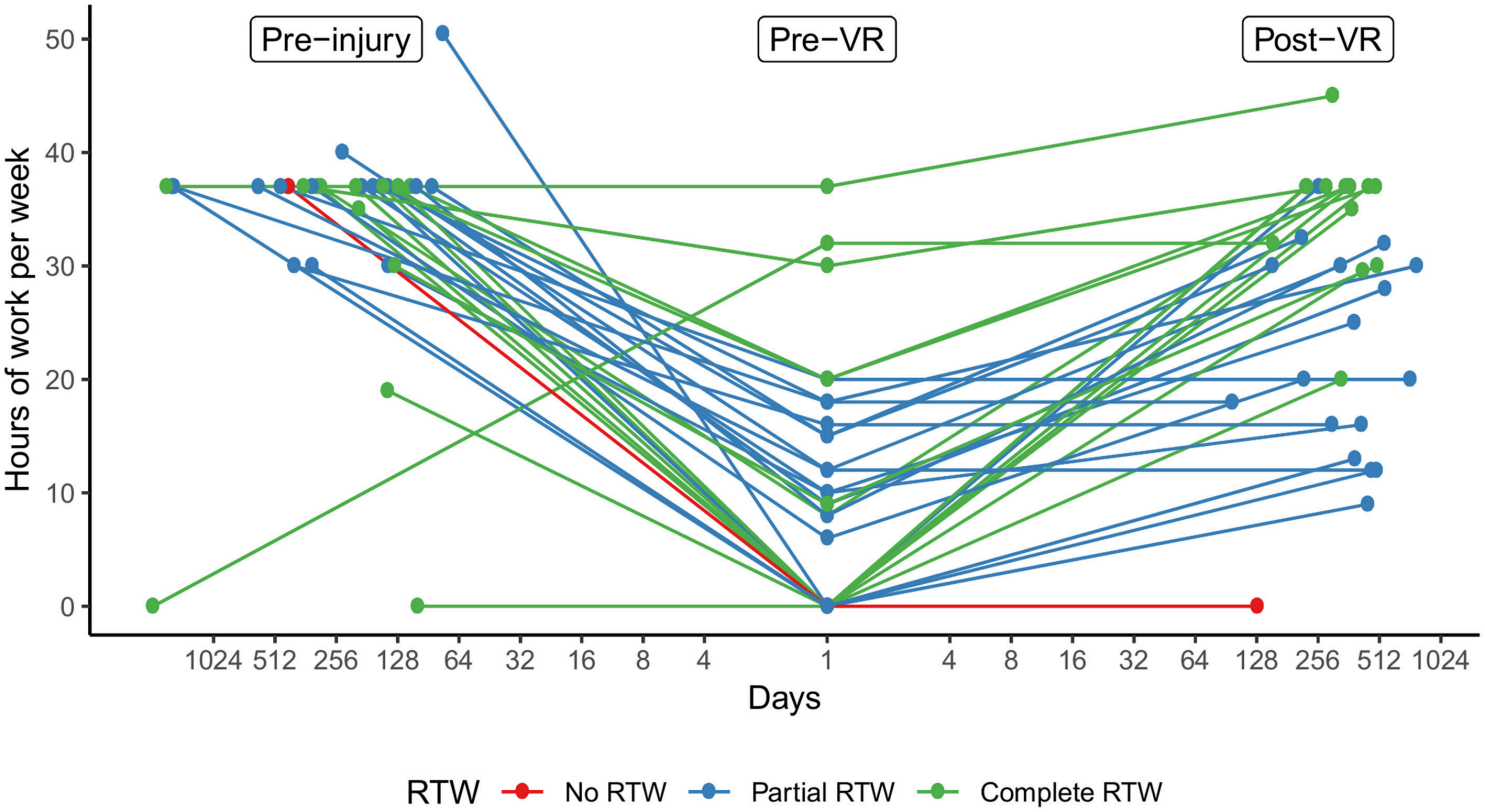
Trajectories of hours at work per week. The graph illustrates each participants' amount of working hours per week on the y-axis at the time of injury (pre-injury; T_1_), at start of VR (pre-VR; T_2_), and after VR (post-VR; T_3_). Time points are distributed on the x-axis by the number of days (log2 transformed) from injury to pre-VR and from pre- to post-VR. Colors indicate RTW-status at post-VR. VR, vocational rehabilitation; RTW, return-to-work.

From pre- to post-VR, mean hours at work per week increased significantly by 17.0 ± 2.2, *p* < 0.001. Each participant either remained or increased the amount of working hours from before to after VR (see Figure 1). That is, no participant worked fewer hours after VR.

**Figure 2:**
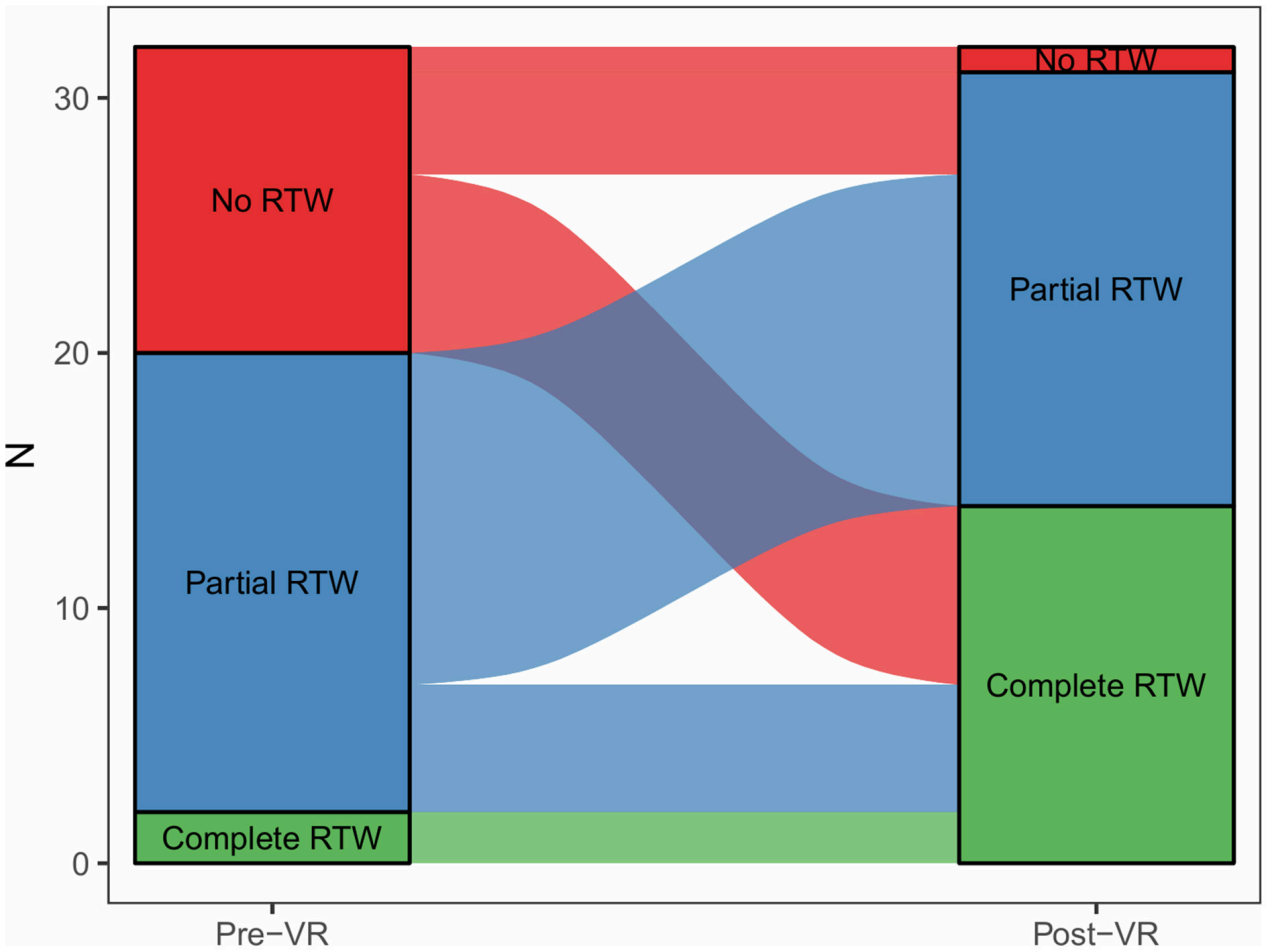
Trajectories of return-to-work status. The graph illustrates participants' development in RTW-status from pre- to post-VR. Streams ending at a higher-level color (0 = red/no RTW; 1 = blue/partial RTW; 2 = green/complete RTW) represent improved RTW-status, streams ending at its own color represent stable RTW-status, and streams ending at a lower-level color would represent regressed RTW-status (no cases of this). Complete RTW represents working the same (or an increased) amount of hours compared to pre-injury, partial RTW represents working fewer hours compared to pre-injury, and no RTW represents not working any hours per week. RTW, return-to-work; VR, vocational rehabilitation.

In terms of RTW, the levels of RTW changed significantly, OR = 14.0, 95% CI [3.5, 55.1], *p* < 0.001, from before to after VR (see Figure 2). Over the course of VR, no participant regressed in RTW-status (e.g., from complete RTW to partial RTW or from partial RTW to no RTW). On the contrary, RTW-status improved for 16 participants (50%) and remained stable for 16 participants (50%). As depicted in Figure 2, the difference in RTW-status was larger between no RTW and partial RTW, *p* < 0.001, than between partial RTW and complete RTW, *p* < 0.51.

#### Predictors of Outcome

Time since injury and sex were significant predictors of change in working hours during treatment (see Table 3). More specifically, double the time since injury was associated with a reduced gain of 5.8 ± 1.4 h, *p* < 0.001. That is, an individual receiving VR at day 100 since injury is observed having 5.8 more working hours per week from treatment compared to an individual receiving VR at day 200. However, participants starting VR in later phases of injury have more time to get back to more hours of work before starting VR, and may thus benefit less from VR, which could explain this association with time. Consequently, we introduced hours at start of VR as a covariate in the model, and the effect of time since injury attenuated from 5.8 to 4.2 ± 1.4, but remained significant, *p* = 0.006. Regarding sex, males had 11.2 ± 5.1 h better effect of treatment compared to women, *p* = 0.035. Age and loss of consciousness were not significant predictors. In terms of RTW, similar but weaker effects of predictors were observed compared to hours at work.

**Table 3:** Predictors of difference in hours at work per week from pre- to post-VR.

	**Hours at work**				**Model parameters**		
**Predictor**	***n***	**range**	***M* (*SD*)**	***Mdn* (*IQR*)**	**Estimate (*SE*)**	***p***	***R*^2^^a^**
**CATEGORICAL VARIABLES**							
**Sex**							
Male	7	14–37	25.7 (10.2)	21 (18)	11.2 (5.1)	0.035	0.140
Female	25	0–37	14.5 (12.2)	13 (14)			
**Loss of consciousness**							
No	25	0–37	16.5 (12.8)	15 (14)	−2.3 (5.4)	0.682	0.006
Yes	7	0–37	18.7 (12.7)	17.5 (15.3)			
**NUMERICAL VARIABLES**							
Age^b^, years					0.01 (0.21)	0.960	<0.001
Time since injury^b^^,^ ^c^, days					−5.82 (1.40)	<0.001	0.364

*Model parameters were estimated by simple linear regression. Response of the linear models was specified as the difference in hours at work from pre- to post-VR. M, mean; Mdn, median; VR, vocational rehabilitation*.

a *For ordinal regression, this value represents the generalized R^2^*.

b *At start of VR*.

c *Log2 transformed. i.e., the effect estimate reflects the change in hours by doubling of the predictor*.

## Discussion

This study described a holistic VR program for mTBI and found that individuals with mTBI had improved employment outcomes after completing the VR program. Time since injury and sex were statistically significant predictors of increase in working hours during treatment.

### Developing a Vocational Rehabilitation Program Within Neurorehabilitation

The holistic approach of this VR intervention can be a complex treatment to learn and conduct, particularly for inexperienced therapists. Thus, a program based on SOPs can act as a tool guiding the clinical reasoning process, by describing the different ways to assess and treat symptoms and the hypothesis of the cause-effect interaction. Moreover, the SOPs will make it easier to disseminate the program to professionals.

The combination of interventions in a multidisciplinary VR program differentiates in nature from more focused interventions such as UPFRONT for mTBI, described by Scheenen et al. (30). UPFRONT is a short intervention involving five sessions of cognitive behavioral therapy aiming at facilitating RTW by enhancing the individual's feeling of competency. Such programs have a clear advantage of being more easily defined, and thereby more easily replicated and adjusted if needed. The Individual Placement and Support (IPS) model, used in other VR studies (31), is another manual-based VR intervention for individuals with brain injury. However, this method involves vocational support only and does not consider other aspects that might influence workability. Given the complexity of mTBI symptoms, it may be important to consider the interaction of biological, psychological, social, and environmental factors (32) in a VR program such as the one described here. LeBlanc and McLachlan (33) further support this view in a study that found an early individualized educational approach to be more effective for employment re-engagement than a general group-based intervention in a cohort of individuals with mTBI.

### Vocational Rehabilitation and Employment Outcomes Following mTBI

During the course of VR, the cohort increased significantly in productive hours per week and improved in RTW-status. Following VR, 97% of participants had RTW compared to 63% before VR. Cancelliere et al. (9) estimated that about 5–20% of workers with mTBI face persisting problems with regard to RTW 1 to 2 years post-injury, and found that research on the prognosis of RTW beyond 2 years of injury is limited. A recent study of 245 adults at 4 years post-mTBI reported that 17.3% had exited the workforce or reduced their working hours compared to pre-injury (11). Another study reported that 59.1% of individuals with mTBI (*n* = 110) returned to work-related activity following a specialized post-mTBI intervention, which was initiated a median of 3.3 months post-injury (34). Comparisons with the present study are challenging, not least due to the variation in time since injury and other baseline characteristics. However, with an average of 2.2 years since injury at completion of VR, these preliminary results indicate that individuals receiving VR has the potential to RTW and improve workability, even 2 years after mTBI. Although not investigated quantitatively in this study, returning to work and resuming former work capabilities may have a substantial impact on the sense of well-being, social integration, and quality of life (12). Thus, further research is warranted on long-term employment outcomes and the effects of VR for mTBI.

Not all participants, who had RTW, returned to pre-injury levels of employment (full RTW). Further, although being a statistically significant change, only half the cohort improved their RTW-status. In some cases, it may be necessary to recommend a reduction in working hours in order to maintain employment and daily life functioning on the long term. In fact, our clinical experience is that returning to pre-injury levels of employment too soon may worsen symptoms and thus be a barrier for maintaining employment to some individuals. Furthermore, the recommendation of graded RTW has been supported in other patient groups (35).

Due to the heterogeneity and complexity of problems after mTBI, we suggest that an important element for outcome success may be the multidisciplinary, holistic, and flexible approach of this VR program, in which the specific contents, intensity and length of the intervention is continuously adjusted to the individual needs of patients. However, this approach makes the program less easily defined and harder to replicate compared to more focused programs such as UPFRONT (30). Thus, a goal for future research could be to investigate the significance of flexibility in VR programs for outcome success after mTBI.

#### Predictors of Employment Outcome

First, results indicated that those starting VR earlier after injury gained more working hours during VR, even when adjusting for working hours at start of VR. There could be several plausible explanations to this relationship. For instance, “the sooner the better” could be a rule with regard to treatment effect or, alternatively, those starting VR later could have more severe or entrenched problems than those starting earlier and thereby benefit less from treatment. How to interpret this relationship is not evident from this study and would possibly require research involving dubious ethical protocols.

Second, we found that men improved significantly more in working hours than women. The reasons for this relationship are unclear, and the results are in conflict with a systematic review finding that sex did not predict RTW following mTBI (9). However, previous research has suggested that women report more post-concussive symptoms than men, although this finding is not consistent (36, 37). Given that women experience more symptoms, e.g., mental health issues such as depression and stress, this could influence RTW and explain why VR was less beneficial for women compared to men in this study. However, we did not have indicators of symptom severity, and further research is needed to provide insight into this matter.

### Generalizability

Since all individuals included in the study were recruited from a single municipality, it is relevant to consider potential biases related to demographics. The included municipality is characterized by a relatively high average income with 55.7% of the population having a higher income than the country's average, and the citizens are highly educated compared to the national average. Thus, the recruitment design of this study introduces a risk of selection bias, and demographics could be influencing the results positively. Further studies would have to investigate whether this VR design applies to individuals from other municipalities with work and education levels closer to and below the country's average.

## Limitations

This study had several limitations. First, the study design was retrospective in nature, the cohort was relatively small, and we did not have a control group. Although results are promising, they are preliminary and do not allow for specific inferences regarding effect of intervention. Although, we adjusted for working hours at start of VR in predictor analyses, the effect of time since injury could reflect spontaneous recovery, and not necessarily a more beneficial effect of VR, in earlier phases of injury compared to later. Second, the cohort was a selected group of individuals with mTBI from one minor community in Denmark. Thus, results should be interpreted with respect to these selection procedures and the fact that results may differ for another population referred to VR under different circumstances. Third, we did not investigate whether participants were able to maintain employment beyond VR. Fourth, no data was available on amount and severity of symptoms.

## Conclusion

In this study, we have initiated the work of defining a multidisciplinary and holistic VR program for individuals suffering from post-concussive symptoms using the SOPs framework. This program will be updated on an ongoing basis in line with its use in clinical practice; however, defining interventions in rehabilitation is an important step toward evidence-based practice and standardized methods. While results of this study are preliminary, both working hours and RTW-status improved significantly with 97% having RTW following VR. Time since injury and male sex were identified as predictors of outcome. In particular, double the time since injury was associated with a reduction of 4.2 h per week. Overall, these results suggest that individuals with persistent post-concussive symptoms may still improve employment outcomes, even years after mTBI. However, further research is needed for any firm conclusions to be drawn regarding the effect of VR, including predictors of effect.

## Author Contributions

FD wrote the first draft of the paper, organized data, performed descriptive analyses, and finalized the paper. TS conceived most part of the data, initiated the study, and had the primary role in describing the content of intervention in the results and discussion sections. MR discussed statistical methods, performed a substantial part of the data analysis, and contributed to description of the methods and results sections. LS is part of the research group and contributed with literature review and supported writing the final manuscript. EF is part of the research group and contributed with discussing the research design and population group, and with writing the manuscript. All authors critically read, improved, and approved the final manuscript.

### Conflict of Interest Statement

The authors declare that the research was conducted in the absence of any commercial or financial relationships that could be construed as a potential conflict of interest.
